# Usefulness of serum hyaluronic acid levels as a predictor of incidence of hand osteoarthritis analyzed by longitudinal analysis from the Iwaki cohort

**DOI:** 10.1038/s41598-021-83693-0

**Published:** 2021-02-18

**Authors:** Tatsuro Saruga, Eiji Sasaki, Ryo Inoue, Daisuke Chiba, Seiya Ota, Hiroki Iwasaki, Ryoko Uesato, Shigeyuki Nakaji, Yasuyuki Ishibashi

**Affiliations:** 1 Department of Orthopedic Surgery, Hirosaki University Graduate School of Medicine, 5 Zaifu-cho, Hirosaki, Aomori 036-8562 Japan; 2 Department of Social Medicine, Hirosaki University Graduate School of Medicine, Hirosaki, Japan

**Keywords:** Predictive markers, Osteoarthritis

## Abstract

The factors predicting hand osteoarthritis (HOA) in patients remain unknown. We aimed to investigate the usefulness of serum hyaluronic acid (sHA) levels in predicting HOA progression from a 6-year longitudinal epidemiological study. A total of 417 participants in the Iwaki cohort were followed-up over 6 years. Hand and knee radiographs taken at baseline and follow-up were scored according to Kellgren–Lawrence grades and Kallman score. Participants were classified into the HOA group and the non-HOA group. sHA levels at baseline were determined by ELISA. Correlations between sHA levels, the number of involved joints, and Kallman score were estimated. Factors related to the incidence or progression of HOA over 6 years were analyzed. The prevalence of HOA was 19.9% at baseline, and 3.6 ± 2.1 joints were involved. sHA levels in the HOA group at baseline were significantly higher than in the non-HOA group (p < 0.001) and correlated with the number of involved joints (r = 0.399, p < 0.001) and Kallman score (r = 0.540, p < 0.001). The incidence rate was 14.5%, and the progression rate was 46.1% over 6 years. Higher sHA levels at baseline were the risk factor of HOA incidence. Thus, sHA levels predicted the incidence of HOA over 6 years.

## Introduction

Hand osteoarthritis (HOA) is a common disease in the elderly, and its prevalence as reported radiographically in population-based studies is 29–89% in the middle-aged^1–5^. HOA causes chronic pain and disabilities that lead to serious problems in activities of daily living. It also has a significant impact on socio-economic status. Although early detection of higher-risk patients is necessary to begin a preventive approach, patients could not recognize the severity of their HOA until it progressed and caused serious pain and disabilities. Moreover, the natural history of this disease and therapeutic strategies for preventing incidence or progress have not been established. While there are several potential problems regarding the high prevalence and progressive activity of this disease, radiographs cannot detect minute changes at an early stage. Hence, an easier quantitative evaluation of disease activity needs to be established.

As the evaluation tool of synovitis, serum biomarkers have attracted attention. Biomarkers are measured from blood and urine, and many substances that specifically reflect the condition of bone, cartilage, and synovitis have been reported^6,7^. Biomarkers are suggested as a diagnostic tool and severity predictors of knee OA (KOA), as well as possibly a prognostic predictor^8^. Among them, serum hyaluronic acid (sHA) is strongly related to symptoms and progression of OA since it reflects the state of synovitis. It is gaining attention as a biomarker for OA severity and a predictor of OA progression. Regarding finger OA, it was revealed that higher sHA levels were correlated with the number of osteoarthritic joints in a population-based cohort study^9^ and progression of joint space narrowing from longitudinal observations focusing on the patients^10^. However, there has been no longitudinal evaluation of the relationship between long-term radiographic changes in HOA and sHA levels in epidemiological studies. It is unclear whether sHA levels could be a predictor of incidence or progress of HOA.

This study aimed to investigate whether sHA levels could reflect the severity and number of involved joints in HOA. Furthermore, we examined the predictive power of sHA levels in determining the incidence or progress of HOA in a longitudinal cohort study. We hypothesized that higher sHA levels at baseline could predict these over 6 years.

## Methods

Subjects were voluntary participants from the Iwaki Health Promotion Project of 2008 and 2014, a community-based program to prevent lifestyle diseases and improve average life expectancy by performing general health checkups and prophylactic interventions^11,12^. It is an annual program that has been performed in the general population living in the Iwaki area of Hirosaki City, located in western Aomori prefecture, Japan, since 2005. This cohort study allows the evaluation of many kinds of diseases and disorders from various perspectives and research into the risk factors of locomotive disability. The study protocol was approved by the institutional review board of the Hirosaki University School of Medicine and performed in accordance with the relevant guidelines and regulations. Informed consent was obtained from all patients before enrollment in the study.

### Subjects

Altogether, 887 volunteers from approximately 12,000 residents participated in this study in 2008. They were recruited via phone calls from public health nurses and an advertisement in the mass media. Those who had renal failure, liver failure, rheumatoid arthritis, malignant tumors, and incomplete questionnaires were excluded from the study. Those who did not undergo radiographic examination were also excluded. A total of 724 participants (273 male and 451 female) were enrolled at baseline. Among them, 408 participants (142 male, 266 female) were followed-up in the Iwaki 2014 cohort. The follow-up rate was 56.3%. Height and body weight were measured, and body mass index (BMI) was calculated.

### Measurement of sHA levels

Blood samples were taken from all participants early in the morning for biochemical examination at baseline and follow-up. Blood sampling was performed before breakfast because circulating sHA increases following a meal^13^. The levels of sHA were determined using the Hyaluronan Assay Kit (Seikagaku Corporation, Tokyo, Japan)^9^. The change in sHA levels over 6 years was defined as ΔsHA.

### Radiographic diagnosis

Radiographs were taken for joint evaluation: postero-anterior view of bilateral hands and antero-posterior view of weight-bearing bilateral knees. The following regions were evaluated from each joint group by trained orthopedic surgeons (RU, HI). The second to fifth distal interphalangeal (DIP), proximal interphalangeal (PIP), thumb interphalangeal (IP), and carpometacarpal (CMC), and scapho-trapezial joints for each hand were graded according to the Kellgren–Lawrence classification (KL)^14^. Radiographic OA was defined as KL grade ≥ 2. Participants with at least one involved joint at baseline were assigned to the HOA group, while those without radiographic HOA were in the non-HOA (nHOA) group. Similarly, the presence of KOA was also evaluated based on the KL scale in both knee radiographs and defined as OA with KL grade 2 or more. Furthermore, the severity of HOA was also scored according to the Kallman scoring systems^15^. Kallman scoring system can semi-quantitatively evaluate the progress of HOA from X-ray images, and is used as an evaluation tool for HOA in various fields such as clinical practice and epidemiology. Individual hand joints were assessed for the presence of osteophytes (graded 0–3), joint space narrowing (0–3), subchondral sclerosis (0–1), subchondral cysts (0–1), lateral deformity (0–1), and the collapse of central joint cortical bone (0–1) with a total of 208 points. To investigate the intra-observer reliability of the scale, 20 randomly selected hand radiographs were scored by the same reader, and two orthopedists (RU and HI) also scored the 20 radiographs to assess the inter-observer reliability. The intra- and inter-observer reliability was assessed by the k-statistic, and they were 0.78 and 0.77, respectively.

In this longitudinal study, participants in the nHOA group with an increasing number of involved joints for 6 years were classified into the incident-HOA (iHOA) group, and participants in the HOA group with an increasing number of involved joints for 6 years were classified into the progression-HOA (pHOA) group. The progression by at least one KL grade of KOA for 6 years was also evaluated and defined as the progression of KOA. Both HOA and nHOA groups were further divided into two groups based on the progression of KOA in each group.

### Statistical analysis

Data input and calculations were performed with SPSS ver. 12.0J (SPSS Inc., Chicago, IL, USA). In the baseline data, Chi-square testing was performed between HOA and non-HOA groups to compare sex, knee OA, and smoking status. The Mann–Whitney U test was performed to compare age, BMI, and sHA levels at baseline. Spearman's correlation coefficients were estimated for age, number of involved joints, Kallman score, and sHA levels. Analysis of variance (ANOVA) and the Tukey method was performed to compare ages and sHA levels in each KL grade of KOA and the presence of HOA and KOA.

In the longitudinal analysis over 6 years, the baseline levels of sHA and ΔsHA were compared using ANOVA and Tukey method among the four groups of the non-HOA group, classified by HOA incidence and KOA progression and the four groups of the HOA group, classified by HOA progression and KOA progression. Furthermore, logistic regression analysis was performed with a model in which the presence of incidence or progression of HOA was a dependent variable, while baseline levels of sHA or ΔsHA and relevant factors like age and presence of knee OA were independent variables. A receiver operating characteristic (ROC) analysis was performed to determine whether the baseline levels of sHA at baseline could predict the incidence or progress of HOA for 6 years. We calculated the areas under the curve (AUC). The optimal cut-off point was the highest Youden index value (sensitivity + specificity − 1). A p value below 0.05 was considered to be statistically significant.

### Ethics approval and consent to participate

The Ethics Committee of the Hirosaki University Graduate School of Medicine approved the study, and all participants provided written informed consent before participation.

## Results

**Figure 1:**
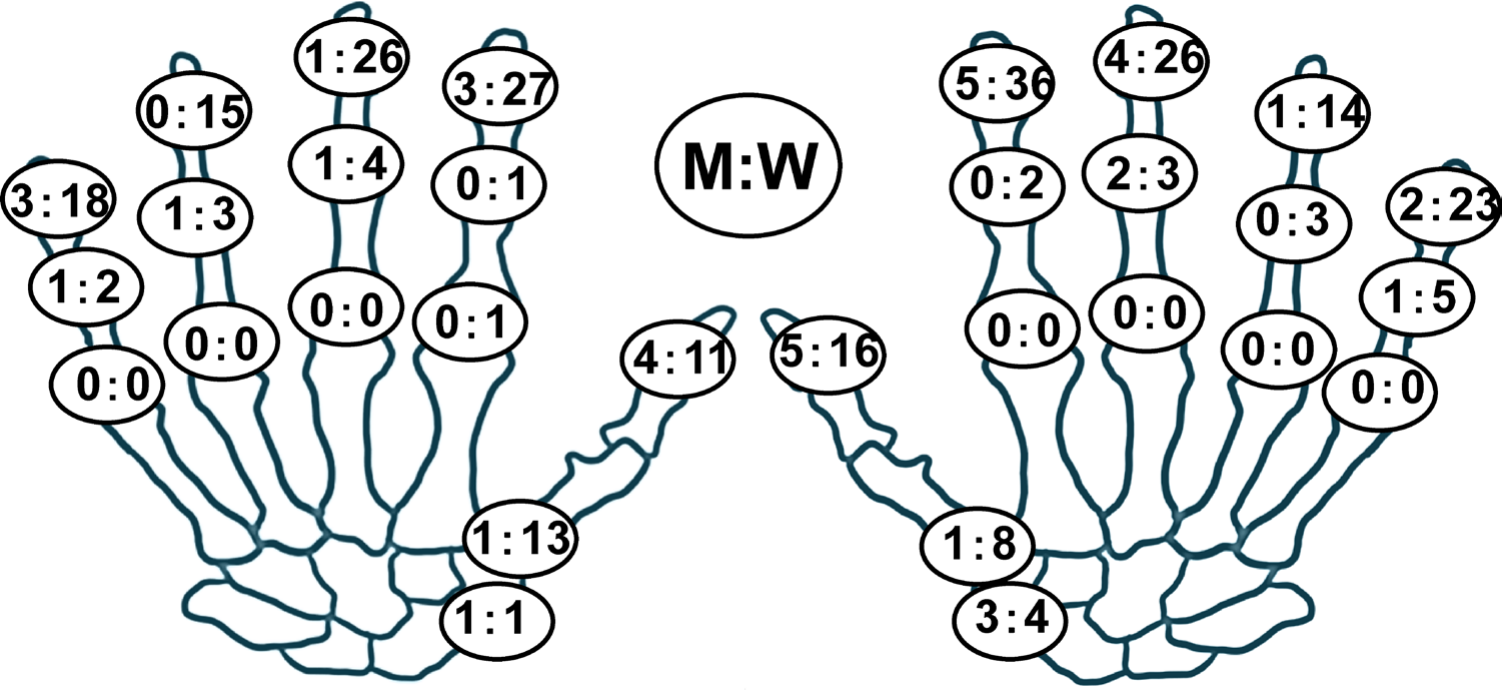
Prevalence (%) of hand osteoarthritis on radiography in each joint in men (M, left) and women (W, right).

**Figure 2:**
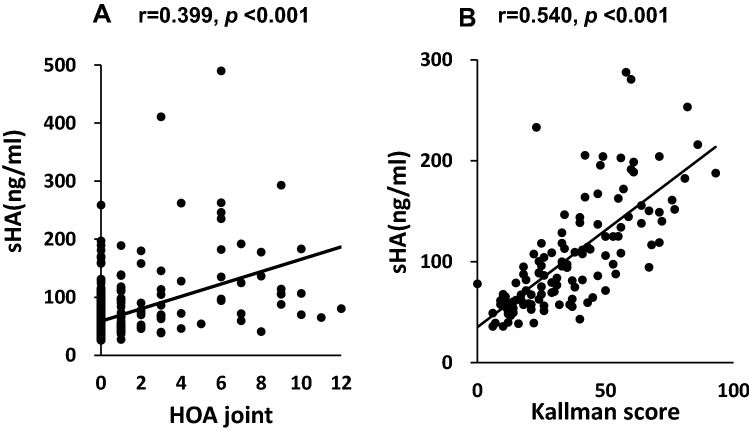
Correlation of sHA levels with the number of involved joints (**A**) and Kallman score by Spearman's correlation coefficients (**B**) at baseline. *sHA* serum hyaluronic acid levels, *HOA* hand osteoarthritis, *r* Spearman correlation coefficient, *p* statistical significance.

Seventy-seven of 408 participants (18.9%) were classified into the HOA group (Table 1). The HOA group was older (*p* < 0.001) and had a higher proportion of females. The prevalence of knee OA was higher (*p* < 0.001), but no significant difference was observed in BMI. The prevalence of HOA at baseline was 10.6% in males and 22.9% in females. Comparing the prevalence of HOA among interphalangeal joints in all cases, the prevalence in the thumb CMC joint, the thumb IP joint, and the DIP joints were high (Fig. 1). The mean levels of baseline sHA were 65.3 ± 35.9 (ng/ml). There was a significant correlation between the levels of baseline sHA and the number of involved joints, and the correlation coefficient was 0.399 (p < 0.001) (Fig. 2A). Similarly, there was a significant correlation between the levels of baseline sHA and higher baseline Kallman score, with a correlation coefficient of 0.540 (p < 0.001) (Fig. 2B). Furthermore, the correlation between sHA levels and age was significant, with a correlation coefficient of 0.631 (p < 0.001) in the non-HOA group and 0.578 (p < 0.001) in the HOA group. In the HOA group, ages were significantly higher with and without KOA. Ages were also significantly higher in participants with KL2 or higher grades of KOA.

**Table 1:** Brief summary of participants.

	Non-HOA (n = 331)	HOA (n = 77)	*p* value (non-HOA vursus HOA)
Age (y)	55.2 ± 9.9	66.1 ± 7.9	< 0.001
Female (%)	61.5	80.2	< 0.001
BMI (kg/m^2^)	22.9 ± 3.0	23.5 ± 3.0	0.071
Knee OA (%)	19.1	57	< 0.001
Smoking (%)	33.4	7.3	< 0.001
sHA (ng/mL)	55.7 ± 27.6	92.3 ± 52.1	< 0.001

Comparison using Mann–Whitney U test and Chi-square test.

Values are given as means ± standard deviations.

p values < 0.05 significant.

**Figure 3:**
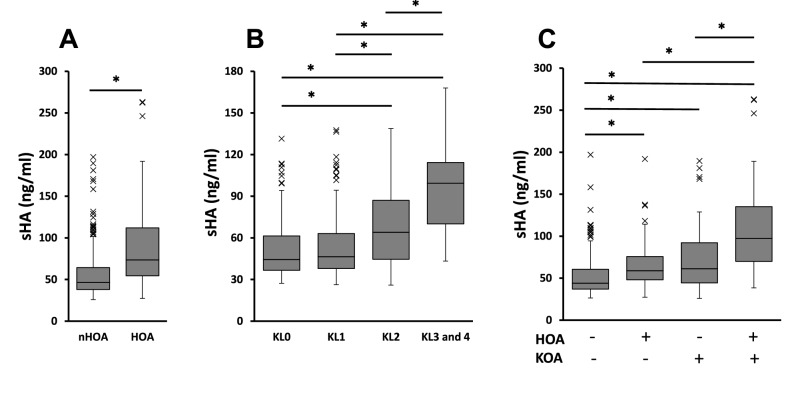
Serum hyaluronan levels among the presence of HOA and KOA, and KL grades of KOA (value ± standard error). P values < 0.05 significant (*) in the analysis of variance (ANOVA) and Tukey method. *nHOA* non-HOA group, *KL* Kellgren–Lawrence grade of knee OA, *KOA* knee OA.

The mean levels of baseline sHA were 55.7 ± 27.6 (ng/ml) in the non-HOA group and 92.3 ± 52.1 (ng/ml) in the HOA group, which was significantly higher than in the non-HOA group (p < 0.001) (Fig. 3A). Levels of sHA were higher below KL grades of KOA (Fig. 3B). The levels of sHA were higher in the HOA group, regardless of the presence of KOA (Fig. 3C).

**Figure 4:**
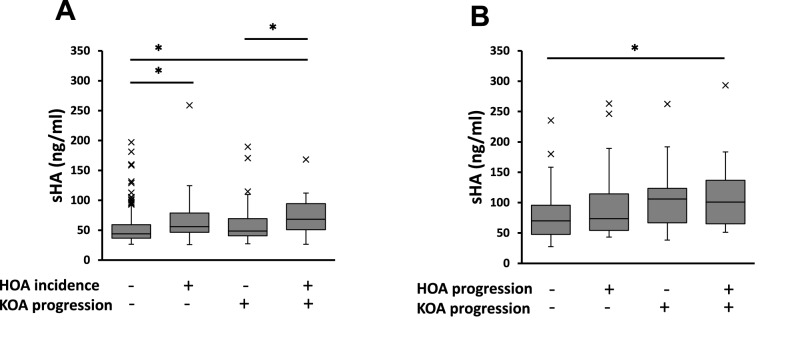
Serum hyaluronan levels among the incidence and progression of HOA and progression of KOA (value ± standard error). P values < 0.05 significant (*) in analysis of variance (ANOVA) and Tukey method.

**Figure 5:**
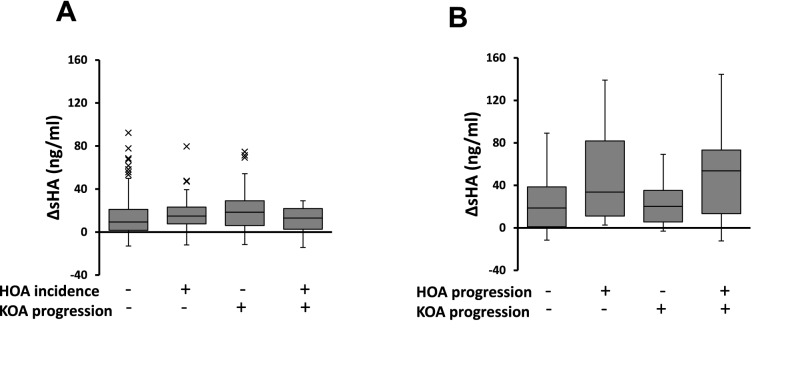
Amount of change in serum hyaluronan levels (ΔsHA) among the incidence and progression of HOA and progression of KOA (value ± standard error). There were no significant changes in the analysis of variance (ANOVA) and the Tukey method.

Over 6 years of follow-up, the incidence rate of HOA was 22.9%, and the progression rate of HOA was 35.0%. The mean baseline levels of sHA in participants with HOA incidence were 66.7 ± 30.2, and higher than participants without HOA incidence (p < 0.001). Levels of sHA in participants with HOA progress tended to be higher than participants without HOA progression; however, there were no significant correlations (p = 0.263). The levels of ΔsHA had no significant correlation with the incidence or progression of HOA. At 6 years follow-up, the non-HOA group was divided into four groups by HOA incidence and KOA progression, and sHA was higher in the participants with HOA incidence, regardless of KOA progression (Fig. 4A). Similarly, the HOA group was divided into four groups by HOA progression and KOA progression. Only without KOA progression, the levels of sHA in participants with HOA progression were significantly higher than participants with none of HOA progression (Fig. 4B). In the levels of ΔsHA, there were no significant differences in each group (Fig. 5).

**Figure 6:**
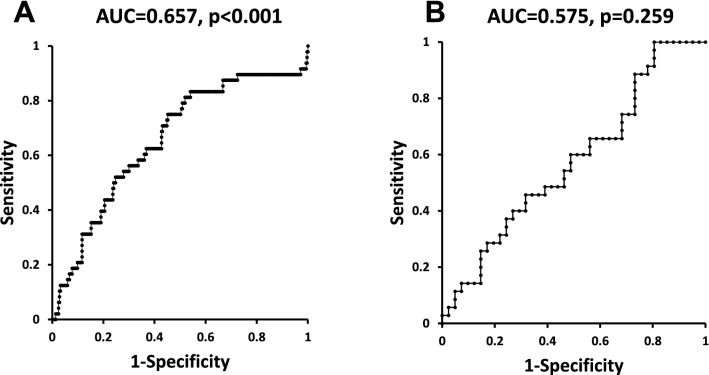
The predictability of incidence (**A**) and progression (**B**) of HOA by the baseline sHA levels in the receiver operating characteristic curve. *AUC* area under the curve, *p* statistical significance.

Logistic regression analysis showed that the levels of baseline sHA were significantly correlated with the incidence of sHA, even considering the effect of KOA (Table2). In the HOA group, the levels of sHA or ΔsHA had no correlation with the progression of HOA (Table3). From the ROC curve, the levels of baseline sHA had a high predictive ability (AUC = 0.657, p < 0.001) for the incidence of HOA in which the cut-off level was 46.6 (ng/ml) (sensitivity = 0.75.0, specificity = 0.45.2) with an odds ratio of 3.63 (Fig. 6).

**Table 2:** Examination of factors related to the incidence of HOA joints.

	sHA				ΔsHA			
	B	*p* value	Odds	95% CI	B	*p* value	Odds	95% CI
Age	0.03	0.066	1.03	0.98–1.06	0.04	0.004	1.04	1.01–1.08
KOA	0.67	0.031	0.51	0.28–0.94	0.721	0.017	0.485	0.29–0.88
sHA	0.074	0.047	1.08	1.00–1.16				
ΔsHA					0.03	0.443	1.03	0.96–1.11

Logistic regression analysis was performed with a model, in which the presence of incidence of HOA as a dependent variable, the baseline sHA/ΔsHA, the relevant factor were age, presence of knee OA as the independent variables. p values < 0.05 significant.

**Table 3:** Examination of factors related to the progression of HOA joints.

	sHA				ΔsHA			
	B	*p* value	Odds	95% CI	B	*p* value	Odds	95% CI
Age	0.07	0.079	0.93	0.86–1.01	0.05	0.174	0.951	0.89–1.02
KOA	1.06	0.075	0.73	0.45–0.92	1.06	0.074	0.88	0.52–0.88
sHA	0.07	0.216	1.07	0.96–1.12				
ΔsHA					0.05	0.286	1.06	0.96–1.16

Logistic regression analysis was performed with a model, in which the presence of the progression of HOA as a dependent variable, the baseline sHA/ΔsHA, the relevant factor were age, presence of knee OA as the independent variables. p values < 0.05 significant.

## Discussion

This is the first population-based longitudinal study to examine the relationship between HOA and sHA levels. From this epidemiological study, it was revealed that, regardless of KOA, sHA levels were higher in participants with HOA and correlated with the number of involved joints and Kallman score. Furthermore, the longitudinal analysis showed that the incidence of HOA over 6 years was associated with the levels of baseline sHA, which meant that higher sHA levels could predict the incidence of HOA in the future. Regarding the relationship between sHA levels and HOA, similar results were obtained in past cross-sectional studies^10,16^, but their validity as a predictor in the longitudinal analysis was not sufficiently investigated.

HA is a glycosaminoglycan found in many joint tissues and an important component of articular cartilage and synovium^10^. It is a marker for synovitis and joint inflammation and is influenced by a variety of factors such as food intake, activity levels, and the presence of disease^17,18^. Therefore, measurement of sHA is performed using blood collected after an overnight fast with less influence of exercise and food. The levels of sHA have been considered a promising biomarker for diagnosing OA and the disease burden^19–21^. Higher sHA levels have been associated with higher KL grades of the knee and hip joints^6,9,22–24^. In HOA, the burden of osteophyte, joint space narrowing, and the number of involved joints were all related to sHA levels^19^. Although the statistical significance of sHA in the HOA group was not demonstrated in the CARRIAGE family study where the association between sHA and HOA was reported for the first time^25^, Filcova reported a significant association with sHA in erosive HOA compared to non-erosive HOA in HOA patients^10^. In normal joints, functional and metabolic activities of hyaluronic acid depend on its high levels and high molecular weight^26^. During inflammation, reactive free radicals from neutrophils in synovial fluid damage and depolymerize HA; this leads to a reduction in its high molecular weight^27–29^. This contributes to a reduction in synovial fluid viscosity and to the dispersion of HA fragments and disaccharide monomers into the circulation^30,31^. Soluble pro-inflammatory cytokines, including interleukin-1 and tumor necrosis factor-α, can also be responsible for the production of HA in synovial fluid^32^. Small HA oligosaccharides in the joint combine with high molecular mass HA and interfere with the normal chondrocyte-matrix interactions^33,34^. They also activate the production and activity of matrix metalloproteinases and nitric oxide synthesis by articular chondrocytes and inflammatory cells^35,36^. Furthermore, monomers of HA can bind Toll-like receptor 2 on macrophages and activate inflammatory responses^37^. This process is involved in the pathogenesis of OA, and it can be inferred that the increased levels of sHA in HOA patients can reflect synovial inflammation and destruction of OA cartilage. Moreover, Chen demonstrated that increased levels of sHA in HOA patients is associated with hand symptoms^25^. However, there is still a lack of sufficient studies analyzing biomarkers in HOA.

We evaluated only knee joints as a confounding factor for this analysis at baseline. Previously, from the perspective of generalized OA, we revealed the sHA levels, which were strongly related to knee and hand OA, from the cross-sectional analysis^9^. Moreover, this study showed that the elderly had osteoarthritis joints in multiple sites even when they were asymptomatic. Ideally, evaluating the presence and severity of OA in more joints would further increase the accuracy of the analysis. However, in epidemiological studies, it would not be feasible and reasonable to perform imaging of multiple sites in view of ethical consideration for exposure to radiation in asymptomatic volunteers and time consumption.

In this study, there was a significant correlation between sHA levels and the number of involved joints in HOA. Furthermore, a correlation between sHA levels and Kallman scores was found. It has been reported that there is a significant correlation between radiological HOA severity and finger pain^3^, and also that serum cartilage oligomeric matrix protein (sCOMP), a type of synovial biomarker, showed association with decreased hand function^16^. In knee OA and hip OA, the association between radiographic severity and sHA has been shown^17,23^. From this study, the relationship between radiographic severity of HOA and levels of sHA was also suggested.

Moreover, it is suggested that the incidence of HOA tended to increase in patients with high levels of sHA, and sHA levels had a predictive ability from the ROC curve. From the Youden index, the cut-off level was calculated as 46.6 (ng/ml). However, this level was lower than the mean levels of sHA in the non-HOA group. Therefore, this cut-off level was considered useful as only a screening tool rather than a diagnostic tool. Filcova reported that a 2-year follow-up study of 88 HOA patients who visited the hospital revealed that Kallman scores increased 2 years later in patients with high levels of sHA^10^. It is considered that the degree of synovitis and cartilage damage may be associated with these correlations. The knee is the largest among weight-bearing joints and has a large volume of cartilage and synovium. Although the individual sizes of finger joints are very small, their number is significant, resulting in large cartilage and synovial volume. Therefore, it seems that association with sHA was also shown in HOA. In our study, the high levels of sHA were correlated with the incidence of HOA. In participants with the progression of HOA for 6 years, Although the baseline levels of sHA tended to be high, they had no statistical correlation with the progression of HOA. The HOA group in this study included 77 participants, and additional follow-up focused on the more samples of the HOA group may show a more detailed association of the levels of sHA with the progression of HOA. It is important to note that symptoms of HOA do not necessarily coincide with radiographic findings. In daily practice, there are older adults who live without pain and activities of daily living (ADL) restrictions, even though their KL grade is high with a significant number of HOA joints, while patients with low KL grades may develop pain and joint swelling and have a great limitation in ADL. Therefore, it is considered necessary to evaluate both symptoms and prognosis when considering the pathology of HOA.

This study has several limitations. First, we did not evaluate the patient-reported outcome scales (PROMs) (for example, Disability of the Arm, Shoulder, and Hand scores or Hand20) and hand function such as grip strength, handedness, pain, and range of motion at the finger joint. We intend to perform longitudinal observations, including PROMs and functional examinations. Secondly, we did not investigate detailed evaluations of erosion in radiographic images. We assessed joints using anterior–posterior radiographs of the hand. Strictly speaking, it may have been better to use lateral views to assess OA in the hand joints^38^. However, in previous cohort studies, the anterior–posterior view was used to assess OA in all the hand joints^1–3,5,39,40^; thus, comparing the prevalence of OA among them might be beneficial. Third, the intake of hyaluronic acid supplements has not been evaluated. Fourth, we did not evaluate the pharmacological therapies that the patients received during the 6 years. Indeed, the uptake of non-steroidal anti-inflammatory drugs or the local injection of steroids in the knee of patients also with knee OA could have reduced the systemic inflammation, with a secondary effect on sHA levels^41^. Fifth, we did not evaluate the hip or other joint OA. The levels of sHA were affected by several OA joints. On the other hand, previous studies have reported that only the knees and fingers were associated with sHA levels^9^. In the future, we intend to analyze the relationship between sHA levels and HOA incidence or progression, including hip and other affected joints prospectively.

Despite these limitations, our results show that the high baseline levels of sHA tend to increase the incidence of HOA. In addition, a significant correlation between the number of involved joints and Kallman score to sHA levels was also seen, supporting the previous report that sHA plays an important role in the pathogenesis of HOA. This study is the first report from a long-term longitudinal epidemiological study of the general population concerning the relationship between sHA levels and HOA.

## Conclusion

Serum hyaluronic acid levels correlated significantly with the presence of HOA, the number of joints involved, and the Kallman score. In the longitudinal study, sHA levels were associated with the incidence of HOA after 6 years, suggesting its usefulness as a predictor of HOA incidence.

## Data Availability

The datasets used and analyzed in the current study are available from the corresponding author on reasonable request.

## Abbreviations

HOA: Hand osteoarthritis
sHA: Serum hyaluronic acid
BMI: Body mass index
DIP: Distal interphalangeal
PIP: Proximal interphalangeal
IP: Interphalangeal
CMC: Carpometacarpal
KL: Kellgren–Lawrence classification
ROC: Receiver operating characteristic
AUC: Area under the curve
sCOMP: Serum cartilage oligomeric matrix protein
